# Privacy by design in systems for assisted living, personalised care, and wellbeing: A stakeholder analysis

**DOI:** 10.3389/fdgth.2022.934609

**Published:** 2023-02-13

**Authors:** Andrea Carboni, Dario Russo, Davide Moroni, Paolo Barsocchi

**Affiliations:** ^1^ Signal & Images Laboratory (SI-Lab), Institute of Information Science and Technologies “Alessandro Faedo”, National Research Council (CNR), Pisa, Italy; ^2^ Wireless Networks Laboratory (WN-Lab), Institute of Information Science and Technologies “Alessandro Faedo”, National Research Council (CNR), Pisa, Italy

**Keywords:** AHA, AAL, privacy by design, ISA, IS

## Abstract

Privacy by design within a system for assisted living, personalised care, and wellbeing is crucial to protect users from misuse of the data collected about their health. Especially if the information is collected through audio–video devices, the question is even more delicate due to the nature of these data. In addition to guaranteeing a high level of privacy, it is necessary to reassure end users about the correct use of these streams. The evolution of data analysis techniques began to take on an important role and increasingly defined characteristics in recent years. The purpose of this paper is twofold: on the one hand, it presents a state of the art about privacy in European Active Healthy Ageing/Active Healthy Ageing projects, with a focus on those related to audio and video processing. On the other hand, it proposes a methodology, developed in the context of the European project PlatfromUptake.eu, to identify clusters of stakeholders and application dimensions (technical, contextual, and business), define their characteristics, and show how privacy constraints affect them. From this study, we then generated a Strengths, Weaknesses, Opportunities, and Threats analysis in which we aim to identify the critical features connected to the selection and involvement of relevant stakeholders for the success of a project. Applying this type of methodology to the initial stages of a project allows understanding of which privacy issues could be related to the various stakeholder groups and which problems can then affect the correct development of the project. The idea is, therefore, to suggest a privacy-by-design approach according to the categories of stakeholders and project dimensions. The analysis will cover technical aspects, legislative and policies-related aspects also regarding the point of view of the municipalities, and aspects related to the acceptance and, therefore, to the perception of the safety of these technologies by the final end users.

## 1. Introduction

Due to the demographic changes among European countries, health and social care have become crucial challenges for many world nations. The general increase of older people compared to the total population affects the current and future economic context. The scientific community has contributed to tackling the problem by studying and proposing solutions and technologies under the so-called *Active and Assisted Living* (*AAL*) (1) and *Active Healthy Ageing* (*AHA*) (2). AAL and AHA aim to propose tools and technologies to improve the ageing process and wellbeing of older people, with particular regard to those in situations of fragility. The broad concept of Active and Healthy Ageing was proposed by the World Health Organization (WHO) as the process of optimising opportunities for health to enhance the quality of life as people age. It applies to both individuals and population groups (3). The definition of AAL comes from the AAL Programme (4), a joint funding activity of partner states of the AAL Association, with the financial support of the European Commission, based on Article 185 of the Treaty on the Functioning of the European Union (TFEU). AAL aims to improve older adults’ autonomy, participation in social life, skills, and employability by providing innovative information and communication technologies (ICT)/digital-based solutions. These solutions, whether products, systems, or services, aim to enhance the older adults’ quality of life, improve the long-term sustainability of health and care systems, and strengthen the industrial base in Europe and internationally. AAL and AHA paradigms typically use systems based on sensors of various types, wearable or contactless, capable of collecting a large number of data to be processed. The analysis of audio–video signals, thanks to the constant growth in performance of signal analysis techniques and related hardware, is increasingly used in AHA/AAL projects. These technologies permit gathering user and environmental information without being invasive directly on the body and provide contactless monitoring capabilities. The new European-level initiative Goodbrothers Cost Action (5) aims to increase awareness of the ethical, legal, and privacy issues associated with audio-based and video-based monitoring. Goodbrothers proposes privacy-aware working solutions for assisted living by creating an interdisciplinary community of researchers and industrial partners from different fields (computing, engineering, healthcare, law, sociology) and other stakeholders (users, policymakers, public services), stimulating new research and innovation. Today, audio- and video-based applications can recognise the general conditions of the individuals (e.g., various activities, behaviour, emotional state, fall detection, food intake monitoring), providing their vital parameters in real-time (e.g., heart rate, respiratory rate) (6). Video cameras and microphones belong to the category of contactless sensors, non-invasive from a technical point of view; nonetheless, their nature makes them difficult to be accepted by end users also for issues related to the perception of privacy. The risk of using these devices is to create a misused surveillance system that might impact users’ lives. From a technical point of view, an approach based on privacy by design is essential to guarantee the security of data and their processing (7).

In this paper, Section 2 contains a state of the art about Privacy by design and Privacy by Default, which have acquired more and more importance following the introduction of the General Data Protection Regulation (GDPR). We will see in detail the requirements imposed by the introduction of the GDPR, concerning European projects, with specific reference to health and audio and video analysis. In Section 3, we will introduce a methodology to correctly define the different stakeholder groups typically part of an AHA/AAL project. We will analyse their specific characteristics or needs by mapping them to the Technical, Contextual, and Business dimensions, allowing us to face the technical, ethical, and regulatory constraints and issues separately and in the best possible way. In Section 4, we will see an analysis of the state of the art of various European projects, in which audio/video analysis plays a fundamental role, grouping them accordingly to the previously defined dimensions. Finally, in Section 5, we will expose, through a Strengths, Weaknesses, Opportunities, and Threats (SWOT) analysis, a set of best practices helpful in introducing the correct identification of stakeholders and application dimensions.

## 2. Privacy and GDPR, a state of the art

*Privacy by design* is a catchword used for the first time in 2000 in a title of a workshop named “Workshop on Freedom and Privacy by design” held at the “Computers, Freedom & Privacy 2000” conference (8). Even if the term was coined around 20 years ago, its meaning could not be precisely identified. The two words *Privacy* and *Design* are abstract concepts that can assume different meanings strictly related to the context scope where they are used (9). Burgoon et al. (10) propose this definition of Privacy: “Privacy is the ability to control and limit physical, social, psychological and informational access to the self or one's group”. This definition makes us see Privacy as a right, freedom, a capacity, a claim, and an ability. *Design,* instead, expresses the intention to establish a set of rules starting from the initial phases of the life cycle of a system. Matching these concepts together, *Privacy by design* refers to finding a meeting point between the formal and legal concept of privacy and the limits of the current information technologies. The design process, in fact, is critical for that privacy and data protection design patterns are applied starting from the project's beginning phases, according to the EU legislation, ensuring privacy and gaining personal control over individuals’ information.

The General Data Protection Regulation GDPR (EU) 2016/679 defines the guidelines for the Processing of Data and their free movement to guarantee the Protection of Information about Natural Persons. The GDPR obligation is addressed to all companies that use, for some reason, information of Natural Persons and not to legal persons (other companies). In addition to harmonising and updating privacy regulations throughout the EU, GDPR aims to redefine companies’ approaches in terms of data protection, mainly because of the continuous and increasingly frequent cyberattacks that companies of all sizes and sectors have been subjected to.

This is GDPR Art.25 recommendation for Privacy by Design (11):

*“Taking into account the state of the art, the cost of implementation and the nature, scope, context and purposes of processing as well as the risks of varying likelihood and severity for rights and freedoms of natural persons posed by the processing, the controller shall, both at the time of the determination of the means for processing and at the time of the processing itself, implement appropriate technical and organisational measures, such as pseudonymisation, which are designed to implement data-protection principles, such as data minimisation, in an effective manner and to integrate the necessary safeguards into the processing in order to meet the requirements of this Regulation and protect the rights of data subjects.”*

### 2.1. Privacy by default

Implementing privacy by default requirements means that once the product or service has been deployed to end users, stricter privacy requirements are already applied automatically. This must be done considering that all sensitive user data must be saved only for the time strictly necessary for their use and no unnecessary data must be requested. Violation of these rules results in a lack of privacy requirements.

The GDPR Article 25 recommendation for Privacy by Default is as follows (11):

“*The controller shall implement appropriate technical and organisational measures for ensuring that, by default, only personal data which are necessary for each specific purpose of the processing are processed. That obligation applies to the amount of personal data collected, the extent of their processing, the period of their storage and their accessibility. In particular, such measures shall ensure that by default personal data are not made accessible without the individual's intervention to an indefinite number of natural persons.”*

### 2.2. Controllers and processors

Reading the GDPR is important to understand the meaning of the terms Controller and Processor, as they are responsible for the application of the privacy and data protection rules and how the services provided by third-party producers must be introduced within a system or project.

#### 2.2.1. Controllers

The following is the GDPR definition of a controller (11):

“*Controller means the natural or legal person, public authority, agency or other body which, alone or jointly with others, determines the purposes and means of the processing of personal data; where the purposes and means of such processing are determined by Union or Member State law, the controller or the specific criteria for its nomination may be provided for by Union or Member State law.”*

Controllers, physically representable by a single individual worker or by a company or legal entity of various kinds, are the persons responsible for the treatment and processing of sensitive data; therefore, they coordinate all the activities that concern them. A Controller may be subject to a legal obligation to process personal data: section 6 of the Data Protection Act 2018 states that anyone who is under such an obligation and only processes data to comply with it will be a controller.

#### 2.2.2. Processors

The following is the GDPR definition of a Processor (11):

“*Processor means a natural or legal person, public authority, agency or other body which processes personal data on behalf of the controller.”*

From this definition, it is clear how the Processors follow the instructions of the Controllers associated with them without having the authority to take important decisions without authorisation, unless the presence of laws that allow it, as specified in the GDPR Article 29. As in the case of Controllers, a Processor can be represented by a single individual worker or by a company or legal entity of various types.

#### 2.2.3. Third-party organisations

Privacy-by-design and Privacy-by-default can also affect third-party organisations: in fact, during the creation of a product, for reasons such as the use of an external product or particular knowledge in certain fields, it may be necessary that an external resource, which can be a company or even a single developer, is included in the process.

The *GDPR* also talks about this possibility (11):

“*When developing, designing, selecting and using applications, services and products that are based on the processing of personal data or process personal data to fulfil their task, producers of the products, services and applications should be encouraged to take into account the right to data protection when developing and designing such products, services and applications and, with regard to the state of the art, to make sure that controllers and processors are able to fulfil their data protection obligations.”*

### 2.3. Pre-GDPR legal framework

In 1992, the integration process to the single European market reached its climax with the Maastricht Treaty and the European Community's creation. However, with a single market, there was also the need to have a European framework law protecting personal data. To this end, the European framework law on the protection of personal data aimed to harmonise rules at the national level and to avoid polarisation: on the one hand, states with regulations that are too lax in attracting companies and investors and, on the other hand, states with rules that are too strict to prevent data from circulating. The EC adopted Directive 46 in 1995, which is now replaced by the GDPR. At that time, data protection was in its infancy. Little was known about it. It was not possible to adopt a uniform standard binding on all States. It was decided to issue a directive to set the objectives but leave room for national legislators. Directives and regulations are two of the most important legal acts of the European Community. However, they are two very different things with essential implications for States: a directive defines objects but leaves each country to adopt national rules to adopt the directive, while regulations are EU laws that are binding in all countries of the EU in a uniform way.

### 2.4. GDPR and health

Although the GDPR has not completely changed the discipline of personal data protection in the healthcare area, it has nevertheless dictated some innovations. The need for its emanation derives from the progressive evolution of the concept of privacy in the light of the continuous changes induced by technological and Information Technology (IT) progress. In a delicate matter such as that of the processing of health data, the European legislator has paid particular attention to protect the right to data protection, which has been elevated to the fundamental right of each individual. As is known, the GDPR is applied directly in EU member states, which have intervened with internal regulatory acts only to repeal any national regulations in contrast with the new discipline and integrate some aspects left to the state's discretion legislator.

Among the significant changes introduced by the regulation, the obligation to keep records of processing activities must be noted, which contain all information related to the processing of personal data carried out by the Data Controllers or, on their behalf, by the Data Processors.

Another important novelty is the introduction of the figure of the Data Protection Officer (DPO). This expert has the task of supervising and facilitating compliance with the regulations on personal data protection. DPO consultation is mandatory for all public health authorities belonging to the National Health Service and for private structures that carry out large-scale data processing; it is not, on the other hand, mandatory for individual health professionals who work as freelance individuals.

In these cases, the processing of health data is considered lawful:

• preventive medicine, diagnosis, social or health assistance, or social services with treatment purposes;
• protection from threats affecting the public health sector, both in the management of services and medical devices;
• to allow advances in scientific or historical research or for statistical purposes.

In all other cases, health data processing requires the consent of the interested party, preceded by appropriate information. Among the information that must be provided to the interested party, the retention time of health data deserves to be reported.

Talking about audio and video recordings, with the introduction of the GDPR, the regulations for declarations of consent have become more stringent: tacit consent is no longer valid but must be given in an informed and unambiguous manner. Before the GDPR, the regulations could vary from country to country depending on the various legislations. In Germany, for example, registration without consent was considered to be punishable by law. In the United Kingdom, concerning the Data Protection Act of 1998 (DPA), this activity was classified as data processing, and it was only necessary to inform end users of the activities being carried out without the need for explicit consent.

## 3. Stakeholders and dimensions mapping

The aspects introduced by the GDPR, data processing and informed consent, are stringent underlying the application of a privacy by design approach. In this article, we want to focus on other aspects, on people, to contextualise their roles and responsibilities. The level of complexity of large-scale projects, with particular reference to EU-funded projects, is very high, and countless factors must be best orchestrated to achieve the pre-established goals. At the basis of the realisation of a project of this type, once all goals have been defined, it is necessary to identify all the stakeholders involved and proceed to arrange them into homogeneous groups. In this phase, it is necessary to pay attention to the end users of the system: the appropriate use of audio/video acquisition and processing technologies mainly involves issues related to privacy, use of data, and perception of safety by end users, which perhaps represents the most difficult obstacle to face for this stakeholder group. This is true especially in the context of AAL/AHA projects, where this group, consisting of older adults, is still not very accustomed to using and understanding technology. To apply the concepts of privacy by default and privacy by design, as desired by the GDPR from the initial stages, it is essential to correctly identify the categories of stakeholders, keeping in mind that, for each of them, the problems related to these concepts will be different and will need to be addressed in a specific way.

Much attention has been given to identifying stakeholders within the work carried out in the European project PlatformUptake.eu project (12). PlatformUptake.eu aims to provide a state of the art regarding open service platforms in the AAL/AHA domains and proposes a valuable methodology and tools to measure the impact and uptake both for existing platforms and for the development of new ones. From PlatformUptake.eu comes the following definition of platform/open platform, which will be used in this paper:

“*A platform is a software system that allows the many-to-many substitutability between applications, services and devices from multiple vendors via common APIs for the benefit of individual users whatever their role is (older person, carer, social worker, care worker, governmental representative, technology developer etc.). It is an open digital ecosystem that connects the individual users to health or social care provisions, to lifestyle and prevention applications and home technology to support their independent living, healthy lifestyles and participation in society. An open platform tries to maximise adherence to the principles of: Open Source, Open Standards-Based, Federatable, Shared Common Information Models, Vendor and Technology Neutral, Support Open Data, Provide Open APIs, Open Usage and Open Adaptation. A Platform is defined as an operating environment under which various applications, agents and intelligent services are designed, implemented, tested, released and maintained.*”

Paragraphs 3.1 and 3.2 definitions come from the work done by the authors in PlatformUptake.eu, which was based mainly on these resources:

• MAST: The Model for the Assessment of Telemedicine—built following seven perspectives of assessment [(1) health problem and characteristics of the application, (2) safety; (3) clinical effectiveness; (4) patient perspectives; (5) economic aspects; (6) organisational aspects; and (7) sociocultural, ethical, and legal aspects].
• OPEA: Open Platform Ecosystem Assessment Framework—a three-dimensional model. The first axis includes the value network of the AAL platform provider, AAL application provider, Health Service or Social Service provider, the informal carers, assisted persons, and the society. The second axis marks the assessment domains of the evaluation: assistance problem and characteristics of the open platform and applications, technical aspects, user perceptions, outcomes, economic aspects, organisational aspects, and contextual aspects. The third axis relates to the three levels of assessing the AAL ecosystem: the platform, application, and service level.
• GLocal Evaluation Framework—the ACTIVAGE reference evaluation framework for AHA Large-Scale European pilots.
• Market Intelligence—also known as business intelligence. It provides several methods to analyse the platforms' maturity and business models:
  ◦ Business Model Canvas, to analyse existing providers' business models.
  ◦ ADL Matrix, for understanding how an industry's maturity and competitive position affects strategy, in terms of industry maturity (from embryonic to aging) and competitive position (from dominant to weak).

### 3.1. Stakeholder groups

The main stakeholders groups identified in the AHA/AAL domain are as follows:

• Primary end users: older persons who benefit from the services provided by the platform.
• Secondary end users: healthcare organisations, home care/community supports, residential care homes, professional caregivers, informal caregivers, and volunteers.
• AAL/AHA solutions developers/providers: hardware manufacturers and software/app developers.
• Authorities and facilitators: public authorities, social security system, insurance companies, and policymakers.
• Open platform providers: EU-funded platforms and commercial open platforms.

The rest of this section will describe the different stakeholder groups and the various dimensions. Finally, we will try to show how they relate to each other.

#### 3.1.1. Primary end user

In the AAL/AHA domain, the primary end user is the individual intended as the main beneficiary of a service or a set of services the considered platform provides. The primary end user directly benefits from these services with an increase of his quality of life. These people can typically benefit from these services directly, for example, by purchasing them from an Open Platform Provider (typically Commercial) or through secondary end users (typically from an organisation such as a healthcare facility or similar). This last scenario is quite typical of EU-funded projects, especially during the test phases, where the secondary end users are also project partners and take care of the selection of the primary end users.

#### 3.1.2. Secondary end users

The secondary end user group comprises care organisations or institutions who contribute to organising, paying or enabling applications and services the platform provides, like healthcare providers, social and wellbeing organisations, etc. Also, the various types of Caregivers are included in this cluster, which contains two subgroups of stakeholders, one represented by the caregiver's family and the other by care organisations. In this document, we refer to the subgroup of care organisations, implicitly considering caregivers as a service they offer. This reflects the typical organisation of a European project in the AHA/AAL domain, in which care organisations recruit caregivers.

#### 3.1.3. AAL/AHA solution developers/providers and open platform providers

The AAL/AHA solution developer/provider group refers to the team of individuals that follow and implement the entire life cycle of the applications, or, more generally, of the products, derived from a given platform. This group includes platform developers, who create and maintain the platform's product services and applications, and third-party developers, who develop standalone applications or products using available Application Programming Interface (API)s and Software Development Kit (SDK). The developer's role in an EU-funded platform is typically very different compared to a commercial one. In the case of an EU-funded platform, most of the people involved from a scientific and technical point of view are researchers. They are an active part of the consortium that submitted the project application to the European community. They, therefore, are aware, even if not always in an in-depth level, of most aspects of the platform, including economic or managerial ones. When we talk about commercial realities, developers are typically employees and often, for security reasons, only aware of the specifics of the sub-projects they work for.

#### 3.1.4. Authorities and facilitators

The authorities and facilitator group includes the public sector service organisers, public authorities, social security systems, insurance companies, municipalities, and policymakers: in general, it can be seen as a larger scale version (regional, national, or international) of the secondary end users cluster. The type of problems involved is very complex aspects also linked to laws, infrastructures, or characteristics such as readiness or the impact they have on a large scale. The main goal is to help citizens to allow them to live an independent life for as long as possible. The first step is the collaboration between the various Health and Care departments and external partners, aiming to research, develop, test, and implement AHA/AAL solutions. It is essential to provide citizens with increased self-reliability and independent living, improving working conditions, increasing efficiency, and improving the municipality's economy. Companies play a crucial role in all this, and therefore the state must support them with not only funds for innovation but also other activities such as periodic exhibits to give visibility to even the smallest companies.

Regarding digital technology's needs and requirements, the two main aspects were “how to relate” and “how to connect.” “How to relate” implies a work that prepares municipalities for each platform type. Here, the main aspects are: the understanding of policies and policy coordination; collaboration on care processes, without which the whole system becomes unstable; information must be understood and defined in its structure and content, while on the application side, it is crucial to connect the systems, infrastructures, in compliance with laws and safety standards. “How to connect” is more about values, citizens having a say, and digital rights and good employership acquire fundamental importance, as does the fact that the profits derived from the use of these platforms must then be redistributed to the society.

### 3.2. Dimensions

In addition to categorising stakeholders, it is essential to identify the application dimensions (or domains) concerning the stakeholder groups. The three specified dimensions are the Technical, Contextual, and Business dimensions.

#### 3.2.1. Technical dimension

This dimension describes and characterises the functionalities and services of a platform, taking into account these fundamental aspects of an Internet of Things (IoT) system:

• device management capabilities: how the devices connected to the services provided by the platform are monitored;
• integration/interoperability: how access to the data and functionality of the services can be made or provided from an API point of view;
• information security (IS): identify and classify possible data vulnerabilities to prevent possible threats;
• types of protocols: types of protocols used, both for the processing and for the transmission of data;
• data analytics: all activities aimed at providing interactive, real-time, predictive, or batch analytics; and
• visualisation capabilities: all activities related to the creation and customisation of Graphical User Interface (GUI)s that show the results of the analysis and allow interaction with them.

#### 3.2.2. Contextual dimension

The contextual dimension is wide and related to all those non-technological aspects of fundamental importance for the realisation of a platform. The main points can be summarised as follows:

• legal and administrative context: regards all legal and administrative issues related to the development and introduction of an AHA/AAL platform;
• ethics and privacy: regards all aspects relating to the processing of data, their type, and how it is reflected on the rights of the end users;
• data sharing and governance: consider the various models (e.g., Citizenship, Economic, Collective, Third-party) and data management; and
• Intellectual Property Register (IPR): concerns various trademarks, patents, copyrights, open or closed access information and services that are exploited in the development or use of a platform.

#### 3.2.3. Business dimension

In this dimension, financial and exploitation aspects are taken into account. It studies the specific business model and includes complex factors of a non-scientific nature that will not be covered in this document.

### 3.3. The analysis and the challenges

For this document, only the two technical and contextual dimensions will be considered, as the business dimension, though exciting and complex, is beyond the scope of the article. The use of audio and video processing technologies brings a series of aspects reflected directly on the technical and contextual dimensions and across the various types of stakeholders identified previously. Above this, all aspects of privacy and data protection imposed by the GDPR and other possible regulations or limitations that may be more stringent (health, legislative, technical, etc.) must be considered.

In this analysis, we will identify with A, B, C, and D the four stakeholder groups defined previously; therefore, we will have:

- Group A: Primary end users,
- Group B: Secondary end users,
- Group C: AAL/AHA Solution developer/provider and Open Platform Providers, and
- Group D: Authorities and Facilitators.

Regarding IS, it is important to define its meaning and its difference from the term cybersecurity. The National Institute of Standards and Technology defines information security as

“*The protection of information and information systems from unauthorised access, use, disclosure, interruption, modification or destruction, in order to provide confidentiality, integrity and availability.”*

In a nutshell, it is about protecting the data of companies, individuals, and institutions, whose confidentiality must be respected, integrity maintained, and availability guaranteed under regulations. The three objectives, in English “confidentiality,” “integrity,” and “availability,” are known by the acronym CIA (13):

- Confidentiality: data are not accessible to unauthorised parties,
- Integrity: data are kept intact and not subject to unauthorised changes, and
- Availability: data are always available to the user who needs these data.

Over time, various researchers have reworked the original “CIA Triad” several times (14, 15). Still, it represents the most precise and recognised way of summarising objectives in the IS field. Physical places where data are stored are computers, mobile devices, hard disks, servers, and, in recent times, cloud environments. Keeping a large amount of information safe is the main objective for those who want to address the issue of information security. In-depth skills in IT security are not required but more in managing data. The defence mechanisms used are many and require specific technologies such as control systems that verify the access of users who want to consult or use them. Another aspect to be taken care of is prevention, starting from the document storage phase and, subsequently, in critical moments such as the transfer from one device to another. The most valuable contents, such as credentials and passwords, must always be kept secret and protected from unauthorised access. Cybersecurity deals specifically with how companies and organisations protect their programs and resources of a purely digital nature.

Unlike what we found for information security, we move into a much more technical field, in which you need to know in depth all the cyber threats you may encounter. Cybersecurity professionals must be able to counter hacker attacks and the appearance of malware. Therefore, it is deduced that while cybersecurity is a problem strictly related to the technical dimension, information security is instead an aspect that borders on the contextual dimension, affecting all defined stakeholder categories. The concept of Information Security Awareness (ISA), therefore, becomes fundamental due to the increase in possible dangerous behaviour of people and the growth of networks and related applications (16, 17). Vroom and Von Solms (18) point out that 48% of security breaches are accidental or related to poor knowledge of IS policies, mainly caused by human errors. The literature shows that the human attitude, intrinsically and especially in people with little technical knowledge, can reveal vulnerabilities and, therefore, contribute to cyberattacks (19, 20). The fact that these behaviours are challenging for an organisation to control leads to the conclusion that ISA training is strongly recommended as an integral part of a company's security policies.

With the increase in the digital transfer of personal data, privacy of the same is a fundamental aspect to be considered when developing services or applications. According to the literature (21), privacy requirements are:

- Anonymity: the inability to recognise a user by third parties or other users,
- Pseudonymity: fictitious names are used to ensure anonymity,
- Unlinkability: the relationships between the subjects and their actions cannot be reconstructed by third parties,
- Undetectability: a third party cannot detect the existence of a component, and
- Unobservability: actions between subjects are hidden.

Especially after the introduction of GPDR, it is critically important that users know what data they are providing, to what processes and for what kind of use, primarily because the use of applications to support daily life is constantly growing. Security awareness training programs, strictly linked to ISA training, aim to facilitate the acquisition and, above all, understand the safety rules to minimise the risk of people harming themselves or the systems they use (22).

A simple analysis can start by identifying the primary activities relating to the two domains taken into consideration and mapping them to the corresponding stakeholders. We will first analyse the technical dimension and then the contextual dimension.

#### 3.3.1. Technical dimension mapping

As far as the technical dimension is concerned, the first group of stakeholders to be considered is Group C, which is responsible for the development intended as the realisation of the hardware/software product that will expose the system's functions. The main challenges for this group of stakeholders are compliance with all the regulations imposed by the GDPR on the treatment and processing of personal and non-personal data, along with the privacy rules and attention to cybersecurity defined in the contextual phase. As regards Group A, it is good practice that there is no relationship between it and the technical dimension, which must be as transparent to the end user as possible, especially if the focus is placed on an application where audio/video acquisition and processing are carried out. The aspects relating to ISA are different and of fundamental importance, as we will see in the next paragraph. Groups B and D can have a technical support role regarding the systems' experimentation, testing, and feedback phases, in particular group B, as it has a close relationship with group A. The other activities mainly concern the contextual dimension.

Knowing notions and being aware of privacy laws is not sufficient for technicians' and software developers to build and set up GDPR compliant products to avoid violations. The observance of some practical guidelines able to combine theoretical aspects with experimental procedures can protect and maintain client and employee personal information and data:

• The collection of data should be kept encrypted and anonymised. For example, alter names, addresses, and other confidential information but taking care to make them however usable for applications and analytics engines.
• The observance of some practical guidelines that combine theoretical aspects with practical procedures can protect and maintain client and employee personal information and data.
• Cloud hosting as an alternative to maintain a physical data centre, especially for smaller firms, can simplify the management of the space for storing and elaborating data, outsourcing security, and compliance controls. However, the choice of an appropriate cloud system is crucial. Data stored in a Cloud can potentially be physically placed in any location around the globe that could be subjected to different privacy laws and security standards, leading to unintentional violations.
• The annual execution of vulnerability assessments using third-party penetration tests and regular vulnerability scans can help identify the system's vulnerabilities even for hackers' newest attack techniques.
• Develop and maintain written information security policy for access control, change management, and data integrity.
• Adopt secure endpoints (firewalls, password and device management, malware and ransomware protection, VPNs, etc.), especially for companies that base their business online in a global market.
• The creation of access management to prevent
  ◦ unauthorised user's access to the system in a way and data, and
  ◦ the verification of access rights to all information resources and to the use of the system.

When using IoT technologies exploited for AHA and AAL purposes, privacy management must consider specific aspects proper of networks, devices, interfaces, mobile applications, etc. Some basic requirements for IoT include the following:

• Identify devices before establishing their connectivity to avoid exchanging data with unauthorised devices.
• All connected sub-systems must be able to interoperate with the main framework controlling things to ensure complete infrastructure control.
• Use specific procedures and tools to provide correct and precise functionality of the components of the network.
• Adopted solutions must comply with Data Protection and IS policies, so that data can be treated with confidentiality, authenticity, and integrity (23).

#### 3.3.2. Contextual dimension mapping

Analysing the contextual dimension is complex and critical in terms of achieving a project's objectives. As previously introduced, the aspects to consider are the legal and administrative context, ethics and privacy, data sharing and governance, and IPR. In this paper, we will analyse the first three aspects and their repercussions on the activities of the various stakeholders. As regards the legal and administrative context, the impact of the GDPR on Groups B, C, and D must be considered. While for Group C the primary regulation to be taken into consideration is the one imposed by the GDPR, which also affects ethics and privacy and data sharing, with regard to Groups B and D, it will also be necessary to take into account other aspects: Group B, speaking about AHA/AAL, will undoubtedly be subject to health regulations that could fall on Group C, as further technological limitations or related to the processing of data could be placed, but also on Group A, in a passive manner. Group D has the same characteristics but presents a more complex scenario as, referring to a municipality, the possible regulations, also according to the specific region of belonging, go beyond the boundaries of this research. Data sharing and ethics and privacy, as seen above, are conditioned by the GDPR for Groups B, C, and D but above all by the rules of IS and the need to satisfy the requirements defined as the CIA triad, i.e., confidentiality, integrity, and availability, and the five privacy requirements: anonymity, pseudonymity, unlinkability, undetectability, and unobservability. These concepts, more familiar to the members of Group C, as naturally connected to technical issues, instead have a contextual counterpart of great importance that can be addressed by introducing the concept of Information Security Awareness within the consortium in favour of all stakeholders groups. Among all these, it is important to dedicate space in this discussion to Group A: one of the main problems of research is to consider its point of view while losing the fundamental one of the users (24). The user perspective is conceived as understanding, and if this is valid for all categories of stakeholders, for Group A, it assumes critical importance because often, the success of a project depends on the level of acceptance, involvement, and understanding by end users.

Regarding acceptance and understanding, the ISA is of great importance: in AHA/AAL systems based on audio and video, the perception of privacy violation by the subjects captured is frequent, and only through a real awareness of how data are protected, transmitted, and processed, it is possible to try to overcome these human limits. The involvement of end users is also an aspect that is often overshadowed. There are various approaches, for example, their participation in the development phases of a project (25) or gamification-type approaches. In the health domain, gamification can be fundamental, educating users to carry out activities aimed at protecting or monitoring their health more enjoyably, thus reducing the concerns related to it (26). Gamification is applied in various sectors and always intends to educate, for example, gamified services help users not to forget to take medicines or to carry out daily exercises. This approach towards services that use audio and video recordings can help ensure a better perception in accepting this type of technology in everyday life: it is educational, maintains users' interest, and supports the protection of health. In addition, this method has been studied in relation to the security domain, specifically ISA (27). A gamification-type approach increases user engagement, but they must be informed of all the privacy aspects that the use of a system of this type entails, such as the type of data used and who has access to it. In this case, developing software with previously identified privacy requirements is essential (28). About user involvement, it has proved to be beneficial at many levels, like improved patient safety, user satisfaction, the reduction in development costs, limiting redesign, and increased likelihood of commercial success (29).

## 4. H2020 projects and audio/video analysis

The goal of this section is to show a state of the art on the most recent AHA/AAL projects in which audio/video analysis has an important role and to show that the classification of stakeholders and dimensions presented in this article can be helpful in the phases of creating a project. The GDPR provides guidelines to follow for the protection and processing of data; however, it considers the technical aspect more than the contextual one. As we will see in this section, in most projects, there is no univocal classification neither for stakeholder groups nor for application dimensions; their existence is often implicit in the nature of a project, but in most cases, it is not dealt with explicitly.

### 4.1. H2020 projects related to audio/video processing in the AHA/AAL domain

Through research carried out mainly with the documentation available on the Cordis Europa portal (30), 25 different projects have been identified, starting from 2013, many of which are currently underway. Their first classification was carried out trying to understand, for each, the contextual or technical dimension of reference (Table 1). Projects focused on the contextual dimension aim to improve aspects such as the uptake, the relationship between the stakeholders involved, the improvement and unification of infrastructures and services, etc. In summary, all aspects fundamental to technological innovation must be accepted and spread in a stable manner. On the other hand, projects focused on the technical dimension aim to create or introduce new and innovative technologies in AHA/AAL research. Every project listed contains both a contextual and a technical dimension. Typically, the ones that focus on the latter need to strengthen the contextual dimension side to create a technology that can be maintained and used over time, even after the end of the projects.

**Table 1:** Projects classification in the contextual and technical dimensions.

Projects in the contextual dimension	Projects in the technical dimension
Platformuptake.eu (31)	Activage (32)
Shapes (33)	Smart Bear (34)
We4aha (35)	Anatomus (36)
Mecasa-ai (37)	Phara-on (38)
Acrossing (39)	Rise-well (40)
Smartwork (41)	See Far (42)
Homes4life (43)	HOLOBALANCE (44)
Ageingatwork (45)	Eyesynth (46)
Ehcobutler (47)	Radio (48)
Gatekeeper (49)	Grow me up (50)
	Enrich-me (51)
	Seizsafe (52)
	IN LIFE (53)
	my-AHA (54)
	Semeoticons (55)

**Figure 1:**
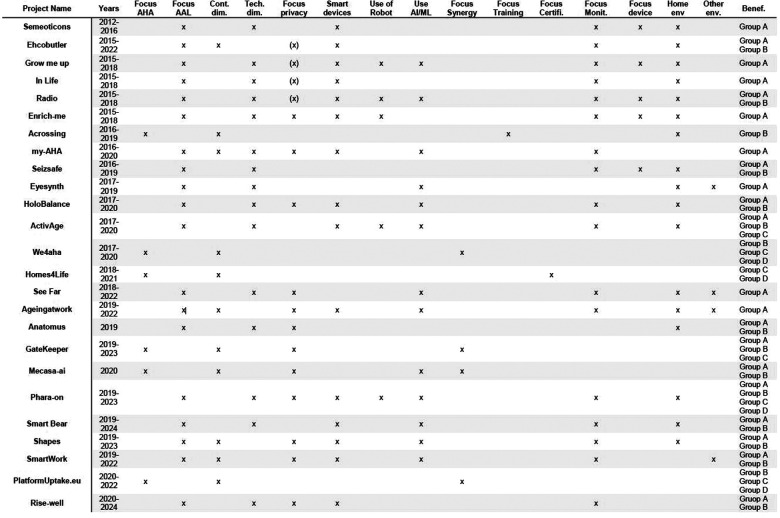
European projects in the AHA/AAL domain. AHA, Active Healthy Ageing; AAL, Active Healthy Ageing.

Our analysis focused on recognising 17 characteristics for each of the projects. Figure 1 shows the results obtained by analysing the aims, methodologies, and results of 25 European research projects.

These characteristics are defined as follows:

• Years: the beginning and ending years of the project;
• Focus AHA: the project is mainly focused on AHA aspects;
• Focus AAL: the project is mainly focused on AAL aspects;
• Contextual dimension: the project belongs to the contextual dimension;
• Technical dimension: the project belongs to the technical dimension;
• Focus privacy: the project implements strategies related to privacy/security aspects. We use “(x)” when the project does not satisfy GDPR directives (i.e. the project is antecedent to the implementation of GDPR rules), “x” when the project satisfies them;
• Smart devices: during the project, have been used or developed smart devices (e.g. smart home appliances);
• Use of robot: during the project, has been used or developed a robot;
• Use AI/ML: during the project, have been used artificial intelligence/machine learning algorithms;
• Focus synergy: the project aims to create synergies and connections among final users;
• Focus Training: the project aims to train final users (e.g. the creation of courses of study);
• Focus certifications: the project aims to create models for the certification of methodologies, products and solutions.
• Focus monitoring: the project aims to monitor users' activities and health status;
• Focus device: the project aims to create a new customised device to monitor, assist, and support final users in their activities.
• Home environment: the project is strictly related to the home environment;
• Other environment: the project is related to other environments (e.g. work environment);
• Beneficiaries: the users that have been recognised as the beneficiaries of the project's output. They are categorised into four groups as previously defined in the paper.

### 4.2. The analysis

**Figure 2:**
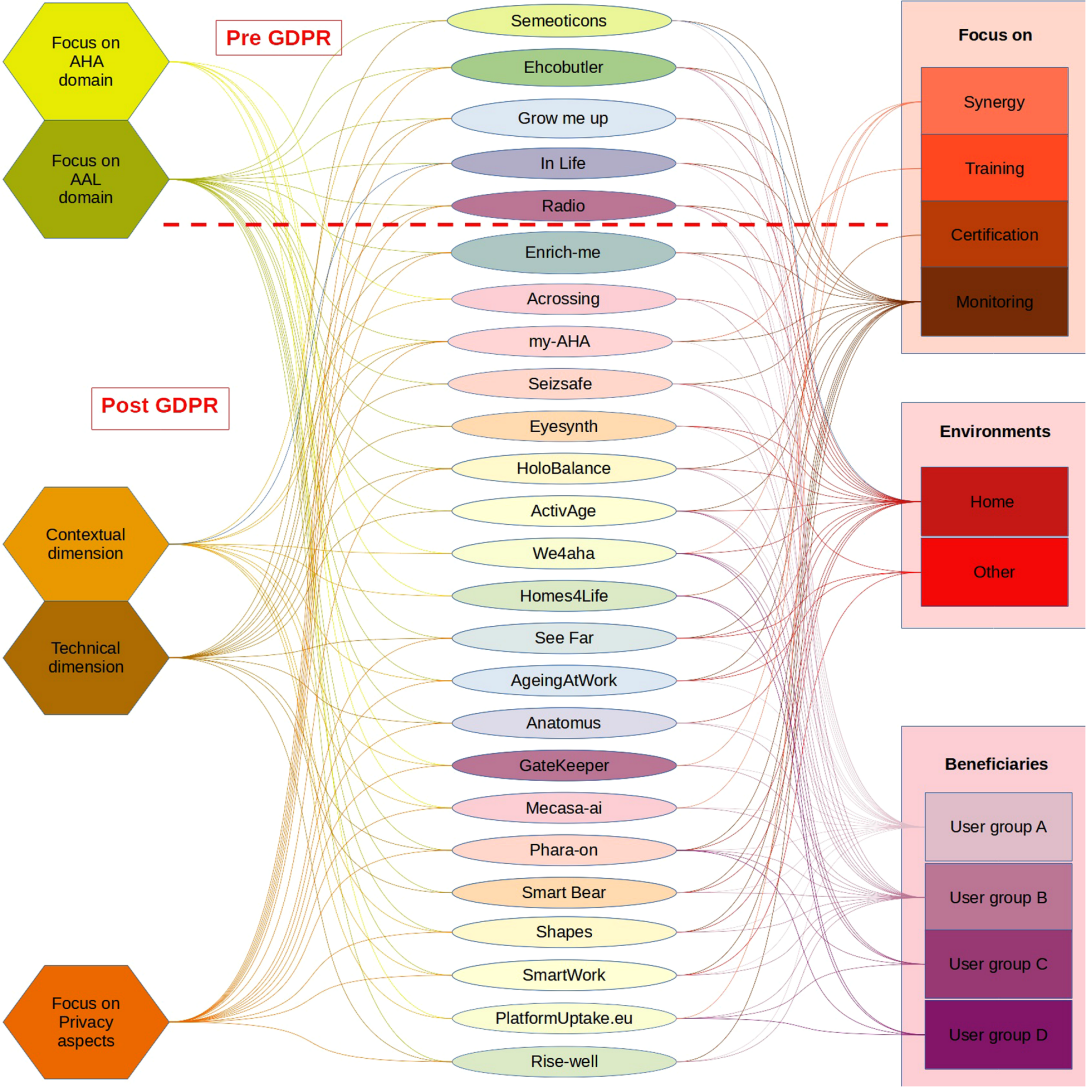
Graphic representation of the characteristic of the projects.

Using Figure 1, we tried to provide a graphical representation of what emerged, visible in Figure 2. This figure has been organised into three columns: in the central column there are, in chronological order and taking into account the year of introduction of the GDPR, the projects examined; in the left column we have collected the intrinsic characteristics (focus on AHA or AAL, focus on technical or contextual dimension, focus on privacy); the right column shows the aims or outputs of the project (the beneficiaries, the application context and the type of application). Looking at the left column, we can see that most of the projects are focused on AAL, since most involve using or developing assistive technologies. The distribution of projects oriented to the technical or contextual dimensions is reasonably balanced with a slight predominance of the former. This information agrees with the previous indication on the majority of AAL-type projects. Since a project always has a contextual dimension, it is essential to know that the project's success also depends on the attention paid to the management of contextual activities. Concerning privacy, there are no significant differences between the phase before the introduction of the GPDR and the subsequent one.

This is partly because the projects are all relatively recent, and each declares appropriate management of sensitive data and the consent of primary end users. From a contextual point of view, the activities are typically limited to the compilation of informed consent without defining formal information strategies (e.g., ISA training). From the technical point of view, regarding the list of requirements specified in Section 3.1.1, the majority pay attention mainly to the correct data management.

Looking at the right column, we can see how most projects focus on home monitoring and have stakeholders from groups A and B as primary beneficiaries. The projects listed, therefore, propose different solutions oriented to the same context. It should be noted that their time windows often overlap, but no synergy between them is highlighted. This prevents the birth or affirmation of standards in the AHA/AAL sector, which unfortunately, to date and despite the multitude of projects and related investments by the European community, do not yet exist. All these different solutions can generate distrust by the primary end users and, therefore, compromise the entire test and data processing phase of a project. It is noted that the definition of stakeholders is often a consequence of the project's aims; hence, the attention paid to Groups A and B but little to Groups B and C is often not even mentioned.

The privacy-by-design approach proposed in this article aims to suggest a unique definition of the four stakeholder groups, their characteristics, and their relationships to ensure consistent and correct data management that is not limited to meeting the written criteria. Each individual must be aware of their role and importance within a project to ensure its success.

The stakeholder groups defined in this article and the two dimensions, technical and contextual, must be modelled and defined according to the project to be carried out. In the next section, a SWOT analysis will be presented, to show Strengths, Weaknesses, Opportunities, and Threats for each stakeholder group and providing guidelines that helps in their definition and in identifying their purpose and boundaries to ensure not only correct data management but also correct data generation.

## 5. Impact on success: a SWOT analysis

SWOT is a useful technique for keeping track of a project's strengths and weaknesses and for analysing and reviewing any opportunities and threats that may appear during its life cycle (56).

Conducting a SWOT analysis can bring significant benefits to a project, such as reducing risks, improving planning, and generally increasing the chances of success. More precisely, the four main points of a SWOT analysis can be defined as follows:

• Strengths: refers to factors internal to the project that should favour its success.
• Weaknesses: refers to factors internal to the project that could make the project fail.
• Opportunities: refers to factors that are external to the project that could make the project successful.
• Threats: refers to factors external to the project that can significantly impede its success. Threats, like Opportunities, are just possibilities, but identifying them allows for alternative plans to be exploited in the unfortunate case they occur.

In this section, we aim to conduct a SWOT analysis concerning the possible impact of the various categories of stakeholders from the point of view of who intends to develop and deploy an AHA/AAL platform, possibly including audio/video data. In particular, we aim to identify the critical features connected to the selection and involvement of relevant stakeholders.

The starting point is the relationships between the Contextual and Technical dimensions and the four stakeholder groups that have been provided in the previous section, intending to highlight the criticality and potential of each of them to achieve the project objectives, with particular regard to the issues of health and audio/video analysis. Through the proposed SWOT analysis, in this section, we will try to summarise the concepts seen previously to make available at a glance the strengths, weaknesses, opportunities and threats of each stakeholder group towards achieving the project objectives. In this analysis, the stakeholder groups covered will be primary end users, AHA/AAL solution developer providers, open platform providers, secondary end users, and authorities and facilitators. We opted to combine the latter two groups in this analysis. Indeed, considering a health facility concerning secondary end users, at the level of SWOT analysis, the scenario is similar, albeit on a smaller scale, compared to that of a municipality if we consider the Authorities and facilitators group. Notice that we conducted the analysis on the basis of the information collected by questionnaires (coming from PlatformUptake.eu's activities) and after extensive discussions with members of the three identified groups. The conducted SWOT analysis does not pretend to be exhaustive. Still, it aims to underline and summarise the key elements most relevant to the community as they emerged from our considerations. To this end, we provide the SWOT analysis in Tables 2–4, respectively, for primary end users, secondary end users (combined with authorities and facilitators), and finally for platform developers.

**Table 2:** SWOT for primary end users.

	Usually positive	Usually negative
	Strengths	Weaknesses
Internal	• Knowledge of expected platform requirements in order to focus on relevant features of AAL/AHA platforms • A good relationship with ICT tools and general technologies • Benefitted from ISA training	• Distrust of technology • Congenital or acquired difficulty (physical or cognitive) that prevents the use of technology
	Opportunities	Threats
External	• Provision of high-quality realistic data to better tune platform services and improve research in AAL/AHA • Creation of a community of users by direct engagement of their peers and word of mouth	• Risk of losing interest • Possibility to provide erroneous data, whether deliberately or not • Risk of dropping out (voluntarily or not)

SWOT, Strengths, Weaknesses, Opportunities, and Threats; AHA, Active Healthy Ageing; AAL, Active Healthy Ageing; ICT, information and communication technologies; ISA, Information Security Awareness.

**Table 3:** SWOT for secondary end users, together with authorities and facilitators.

	Usually positive	Usually negative
	Strengths	Weaknesses
Internal	• Capability to represent the “market pull” in AAL/AHA platforms • Skills in providing ISA training to linked primary users of AAL/AHA platforms • Previous skills on AAL/AHA topics • Possession of an active and lively network of primary end users	• Inefficiencies in the internal process • Lack of flexibility in reorganising the management of care with the related primary users • Distrust of changes • Possible perception of an increased workload during the introduction of services provided by the AAL/AHA platform
	Opportunities	Threats
External	• Creation of a community of secondary end users by networking or clustering activities (e.g., by participation in associations) • Collection of data with high reference value concerning the sustainability in using the AAL/AHA platform • Creation of new highly professional profiles inside the institutions	• Search for primary end users for the test phases is approximate, and the profiles chosen are not suitable • The relationship established with the network not very profitable • Possible distrust of the primary end users towards authorities might jeopardise the positive effects introduced by the AAL/AHA platform

SWOT, Strengths, Weaknesses, Opportunities, and Threats; AHA, Active Healthy Ageing; AAL, Active Healthy Ageing; ISA, Information Security Awareness.

**Table 4:** SWOT for platform developers.

	Usually positive	Usually negative
	Strengths	Weaknesses
Internal	• Capability to bring the “technological push” into AAL/AHA platforms providing innovative service in a more ample response to users’ requirements and market pull • Good predisposition towards open-source platforms and integration of services • Sensibility vs. privacy by design and privacy by default paradigms	• Possibility of having scarce ISA training • Difficulties in understanding users’ needs and the number of actors and complex interactions needed in a AAL/AHA platform • Scare flexibility in customising the platform to adequate to slightly different sets of groups
	Opportunities	Threats
External	• Development of high skilled professional figures for the generation and maintenance of AAL/AHA platform • Cross-fertilisation with platforms in other domains, including home automation, artificial intelligence and audio/video services	• Loss of interest in the development due to the possible scarce impact of the platform in its beginning stages • Possibility of losing key persons during critical, crucial implementation steps due to the job market • Difficulty in hiring adequate resources

SWOT, Strengths, Weaknesses, Opportunities, and Threats; AHA, Active Healthy Ageing; AAL, Active Healthy Ageing; ISA, Information Security Awareness.

For primary end users, strengths and weaknesses correlate to the capabilities in interacting with an ICT platform and the issues of different nature that might impede such familiarity with technologies. In principle, every AAL/AHA service should be designed to be accessible to everyone, e.g., by espousing universal design principles. However, the suggestion is to carefully balance the primary end users group to be included, selecting for the first test phase a limited number of testers having a good relationship with ICT tools. At the same time, in subsequent stages, the groups can be enlarged, considering users exhibiting weaknesses or other frailties. Opportunities depend and are proportional to the size of the groups of primary end users involved, granting real data sets for validation of platforms and community building. Threats exist and should be appropriately mitigated. For instance, the risk of losing interest might be counteracted by introducing gamification approaches described earlier in this paper.

The SWOT analysis for secondary end users reports similar considerations, although at a different scale. For instance, the adaption of the platform can be favoured inside associations of secondary end users (e.g., associations of municipalities in a region or, again, groups of nursing homes). It is vital to notice that selecting proper secondary end users can bring into the deployment of the platform knowledge about the “market pull.” This is an essential point in revising business plans and in focusing the platform on those services that are helpful and sustainable. Proper validation of sustainability is a possible by-product achievable as an opportunity by running pilots with secondary end users. Vice versa, weakness should be sought in potential organisational inefficiencies and scarce inclination to change, possibly due to the fear of additional workloads or the management of different procedures.

Finally, for platform developers, internal characteristics are linked to developers' personal capabilities and sensibilities and to features that cannot be taken for granted by default even in this group of stakeholders, e.g., ISA training. In response to the users' requirements and the market pull, it is expected that developers can act proactively by providing a technological push. External issues are linked to the general job market and to the difficulty of carrying out a project at a steady pace. However, the possibility offered by the job market favours cross-fertilisation and can help bring leading-edge technologies in the AAL/AHA domain.

## 6. Conclusions

This article analysed the privacy-by-design requirements of a system for assisted living, personalised care, and wellbeing, particularly reviewing projects that provide audio and video signal processing. This analysis was carried out to understand how the different categories of stakeholders involved in the realisation of a project influence or are influenced by the privacy requirements. The study of these requirements started from the description of the regulation imposed by the GDPR. Subsequently, the categories of stakeholders and the dimensions were introduced and related to each other to show their importance in a privacy-by-design approach. Afterwards, we conducted a review of recent European AHA/AAL projects in the audio/video domain. This section represents not only a state of the art but also an opportunity to highlight how the concept of technical and contextual dimensions is important and often not consciously addressed, as well as the correct stakeholder recognition. The article concludes with a SWOT analysis carried out on the main categories of stakeholders identified, which can be helpful to face the setup of a project or the analysis of one in progress in a conscious way, so that the regulations imposed by privacy-by-design and GDPR do not become risk factors that compromise the success of the activities.
